# Zishenwan Decreases Kidney Damage in Recurrent Urinary Tract Infection through the Inhibition of Toll-Like Receptor 4 Signal

**DOI:** 10.1155/2018/5968657

**Published:** 2018-11-05

**Authors:** Guoqiang Liang, Hua Tang, Daolei Ni, Yan Ren, Chenxi Zhang, Wenyi Zheng, Yongliang Song, Xiaofeng Shen, Weimin Jin, Chunbo Jiang

**Affiliations:** ^1^Suzhou Academy of Wumen Chinese Medicine, Suzhou 215000, China; ^2^The Suzhou Affiliated Hospital of Nanjing University of Chinese Medicine, Suzhou 215000, China; ^3^Second Military Medical University, Shanghai 200433, China; ^4^Department of Nephrology, Suzhou Hospital of Traditional Chinese Medicine, Suzhou 215000, China; ^5^Department of Orthopaedics, Suzhou Hospital of Chinese Medicine, Suzhou 215000, China

## Abstract

**Objectives:**

To investigate the mechanism of the protective effect of Zishenwan on pyelonephritis rats.

**Methods:**

In the rat model of pyelonephritis, protective effects of Zishenwan, the content of secretory immunoglobulin A (SIg A), and interleukins were detected by ELISA. The expressions of TLR4-NF*κ*B pathway were detected by Western blot in renal and urinary tract mucosa. The protective effect and influence on TLR4-NF*κ*B pathway of Zishenwan were studied.

**Results:**

Zishenwan protected rats from pyelonephritis which related to the increase of SIgA, the regulation of interleukins, and the inhibition of TLR4-NF*κ*B pathway. Serum containing Zishenwan can significantly decrease LPS-induced expression of TLR4, MyD88, and NF*κ*B* in vitro*. And the inhibition of TLR4 signal by Zishenwan related to the degradation of TRAF3 and TRAF6.

**Conclusions:**

Zishenwan protected rats from urinary tract infection by clearance of bacteria and decrease of tissue damage. 20S proteasomes mediated the degradation of TRAF3 which is important to the decrease of tissue damage from Zishenwan.

## 1. Introduction

Urinary tract infection (UTI) is a common infectious disease in the general population [1]. According to the report, about 1/3 of adult women have had UTI in their lives, and 27%-28% of healthy women had UTI more than once [2, 3]. Current therapies for urinary tract infection are based on the administration of antibiotics [3]. When UTI affects patients with low immune function, antibiotics might be used much longer and more frequent [3]. In this state, fungal infection, drug resistance, and dysbacteriosis associate and bring many difficulties for the clinical treatment of urinary tract infection, and recurrent UTI induces pyelonephritis. As a reason, enhancing systemic immunity and mucosal immunity should be important to prevent and control urinary tract infection, especially the recurrent urinary tract infection [3]. Traditional Chinese medicines are widely used in the therapy of urinary tract infection, especially the recurrent urinary tract infection in Asia [4, 5].

In traditional Chinese medical science, recurrent UTI belongs to the category of “Lao Lin" and “deficiency of kidney and bladder heat” is the pathogenesis of recurrent UTI. “Zishenwan" comes from the “LanShiMiCang”, and it is an effective drug treating “Lin syndromes” for a long time. Some components in Zishenwan have antimicrobial activities and it might be an important factor in the treatment of recurrent UTI [6–8]. But according to the observation of clinic, Zishenwan improves the clinical symptoms and the cure rate of UTI, and it associates with the upregulation of CD3^+^T cell number and ratio of CD4^+^/ CD8^+^ T cells. So Zishenwan has protective effects other than the direct antimicrobial activity.

Mucosal immunology is an important mechanism in protecting infection [9, 10]. It is achieved by a combination of factors and induces specific immune responses involving cellular and antibody response [11, 12]. The major antibody at mucosal surfaces is secretory IgA (SIgA) which binds antigens and prevents their adhesion and adsorption to the surface of epithelial surface and eliminates these antigens [13, 14].

TLR4 signaling pathway plays important roles in the development of immune diseases in clinic [15]. During the pyelonephritis, lipopolysaccharide (LPS) coming from uropathogenic* Escherichia coli *(UPEC) can be identified by TLR4 and induce cytokine production through MyD88-NF*κ*B pathway in renal tubular epithelial cells [16, 17]. Meanwhile neutrophils trigger the innate immunity [18]. Therefore, regulation of the TLR4 signaling pathway influences the immune balance and cell death and it is important in clinical trials of pyelonephritis.

In our experiments, we observed beneficial effects of Zishenwan in chronic pyelonephritis rats and these beneficial effects might come from the secretion of SIgA in the urethral mucus and inhibition of TLR4 signal in rat bladder smooth muscle cell. Our results showed a novel mechanism of Zishenwan in the treatment of recurrent urinary tract infection.

## 2. Methods

### 2.1. Animals

Male SD rats (180-220 g) were supplied by JOINN Laboratories (Suzhou); license number is scxk (苏) 2013-0003. All surgical procedures and care administered to the animals were approved by the institutional ethic committee, and this study also complied with the criteria in Guide for the Care and Use of Laboratory Animals of Suzhou Hospital of Traditional Chinese Medicine.

### 2.2. Rat Model of Chronic Pyelonephritis

Rat model of chronic pyelonephritis was established following the protocol of Goluszko et al. After fasted for 24 hours, penises of rats were bound with thin thread after fasted for 18 hours. Then 0.5ml of* Escherichia coli (E.coli) O111B4 *(10^8^/ml, it is provided from Suzhou Center for Disease Control and Prevention) was injected into the bladder and the thread on penis was released after 24 hours. The model of chronic pyelonephritis was evaluated after 6 weeks.

Rats which were induced with chronic pyelonephritis successfully were divided into 3 groups randomly; model group (10ml pure water/kg), Zishenwan group (27.7g crude drug/kg), and Levofloxacin group (0.23g/kg). Untreated rats were used as control group and were administrated with pure water (10ml/kg). 8 rats per group and they were administrated for 30 days. After the last administration, rats were fasted for 18 hours and anaesthetized with pentobarbital sodium. Then, urine and kidneys were collected.

### 2.3. Histological Analysis

Tissues from mice were fixed in 4% paraformaldehyde, dehydrated for 12 hours, embedded in paraffin wax, cut into 3*μ*m-thick slices, and then examined via light microscopy (Olympus, Tokyo, Japan) for hematoxylin-eosin (HE) immunohistochemistry.

### 2.4. Cell Line and Culture

Rat bladder smooth muscle cell is a gift from China Pharmaceutical University and cultured in complete medium (DMEM containing 10% FBS and 1% Pen/Strep all from Gibco, CA, USA).

### 2.5. Zishenwan Serum

15 male SD rats were randomly divided into 3 groups: normal control serum group, low dose serum group, and high dose serum group (n=5). Rats from low dose serum group were administrated with Zishenwan (5.54g crude drug /kg) daily and rats from high dose group were administrated with Zishenwan (27.7g crude drug /kg) daily. Rats from normal serum group were administrated with saline: the rat body surface area ratio is converted into the equivalent to clinical dose, respectively, to 1, 5 times the amount of medication, and normal serum group were given equal volume of saline, with continuous administration for 7 days. Serum was inactivated at 56°C for 30 min and sterilized by filtration; then it was stored at -80°C. The drug serum and serum free DMEM culture medium were allocated according to the ratio of 1:9, and the culture medium containing 10% serum was obtained.

### 2.6. ELISA

Immunoglobulin A (IgA) in the urine was measured using Rat Immunoglobulin AELISA Kit (CUSABIO and CusAb, CSB-E07987r, Baltimore Avenue, MD). Collected urines were centrifuged at 5,000g for 15 min to remove insoluble matters, and the supernatant was used for ELISA assay. The level of SIgA was assessed by ELISA according to the protocol of the supplier.

Interleukins in the urethral mucus were measured using ELISA kit (eBioscience, San Diego, CA). The urethral mucus was separated carefully and weighted. Then, the urethral mucus was powdered into liquid nitrogen and mixed with PBS (50mg/ml). The mixture was violently shocked at 4°C and centrifuged at 17,500g for 15 min to remove insoluble matters. Levels of IL-4, IL-6, and IL-10 in supernatant were assessed by ELISA using monoclonal antibodies and the procedure recommended by the supplier.

### 2.7. Cell Apoptosis Assay

The apoptotic cells were quantified using the annexin V and PI double staining kit (BD Biosciences). Briefly, cells were collected, washed with PBS, and resuspended in 200 *μ*L binding buffer containing 5 *μ*L annexin V (10 *μ*g/mL) and 10 *μ*L PI (20 *μ*g/mL) for 30 min. Then samples were immediately analyzed using flow cytometry.

### 2.8. Western Blot Analysis

Rat bladder smooth muscle cells and homogenate treated samples of tissues were lysed in RIPA (Beyotime, Shanghai, China) and the protein concentration was determined by a Bradford kit (Beyotime, Shanghai, China). The proteins were treated with SDS sample buffer at 100°C for 10 min and resolved by SDS-polyacrylamide gel electrophoresis (10% acrylamide) and transferred to polyvinylidene fluoride (PVDF) membrane. Membranes were blocked with PBS containing 3% bovine serum albumin (BSA) for 1h at room temperature. Following incubation with the primary antibodies specific for *β*-Actin (1:2,000, PR-0255, Zsbio, Beijing, China), TLR4 (1:2,000, ab22048, Abcam, Cambridge, MA), MyD88 (1:1,000, ab2064, Abcam, Cambridge, MA), NF*κ*B/p65 (1:1,000, ab16502, Abcam, Cambridge, MA), p-I*κ*B*α* (Ser32/Ser36) (1:500, CY6280, Abway, Shanghai, China), I*κ*B*α* (1:1000, ab32518, Abcam, Cambridge, MA), TRAF3 (1:1000, ab36988, Abcam, Cambridge, MA), and TRAF6 (1:1000, ab33915, Abcam, Cambridge, MA) at 4°C overnight, the blots were washed in PBS containing 0.1% Tween and incubated with secondary antibodies (1:2,000, ZDR-5306 or ZDR-5307, Zsbio, Beijing, China) for 1h at room temperature. Blots were then washed four times with PBS containing 0.1% Tween. Immunolabeling was detected by enhanced chemiluminescence.

### 2.9. Statistical Analysis

Results were expressed as mean ± standard deviation (SD). The data were analyzed by one-way ANOVA analysis of variance followed by Dunnett's test. Statistical differences were considered statistically significant when* p*<0.05.

## 3. Results

### 3.1. Zishenwan Decreased the Number of Bacteria and Tissue Damage in Rat Model of Chronic Pyelonephritis

**Figure 1:**
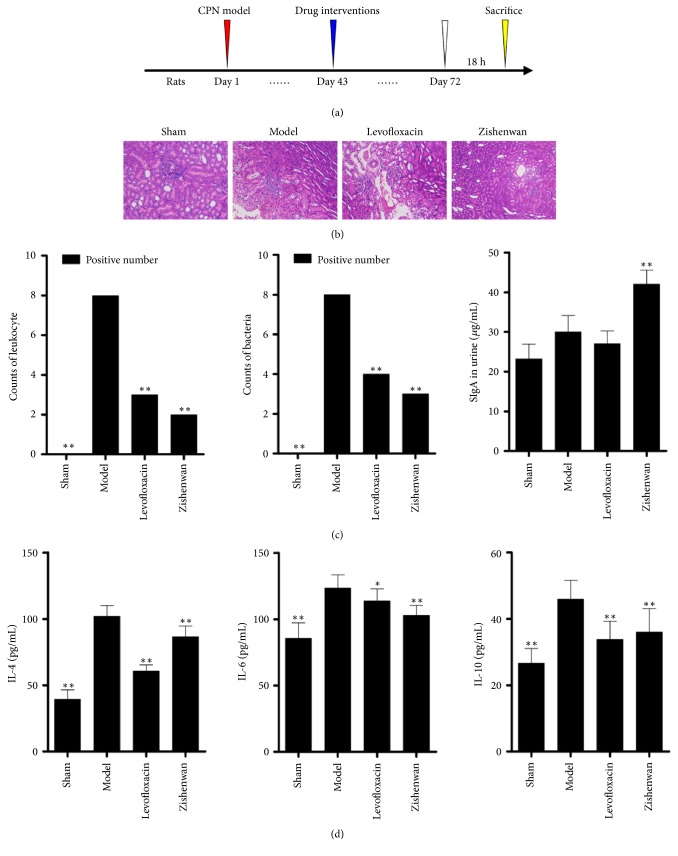
Administration of Zishenwan decreased tissue damage in rats of chronic pyelonephritis model. (a) The design of the experiment* in vivo*. (b) HE staining shows that administration of Zishenwan decreased tissue damage in renal pelvis mucosa of model rats. (c) Administration of Zishenwan significantly decreased the infiltration of leukocytes and bacteria number on renal pelvis mucosa. SIgA in urine were also increased after the treatment of Zishenwan. (d) Treatment of Zishenwan decreased the production of IL-4, IL-6, and IL-10. *∗*, p<0.05 compared with model group; *∗∗*, p<0.01 compared with model group.

From the results of HE stain, infiltration of a large number of inflammatory cell, interstitial fibrous tissue hyperplasia and fibrosis, telangiectasia, interstitial fibrous proliferation, telangiectasia, interstitial hyperemia and edema, necrosis, and loss of epithelial cells of renal pelvis mucosa were observed in model group (Figure 1(b)). The administration of Levofloxacin rescued pathological changes (Figure 1(b)) and decreased infiltration of inflammatory cell and bacteria number (Figure 1(c), p<0.01 comparing with model group). In Zishenwan group, infiltrations of inflammatory and bacteria number were also decreased and they were similar to the Levofloxacin group (Figure 1(c), p<0.01 comparing with model group), but the structure changes of glomerular and the renal interstitial fibrosis were normalized comparing with the model group and the Levofloxacin group (Figure 1(b)).

Components from Zishenwan were reported to inhibit the growth of bacteria, such as berberine and cinnamaldehyde, but beneficial effects of Zishenwan in pathological changes were much more significant. So we hypothesized that factors other than direct antibiotic activity should contribute to the treatment effect of Zishenwan. At first, we detected the secretory immunoglobulin A (sIgA), an important antibody relating to mucosal defense, and we found the concentration of sIgA increased in model group, Levofloxacin group, and Zishenwan group (Figure 1(c), p<0.05, comparing with sham group). But Zishenwan induced higher secretion of sIgA comparing with model group and Levofloxacin group (Figure 1(c), p<0.01, comparing with model group). We also detected interleukins relating to immunoreactivity, and we found the increase of IL-4 and IL-6 and the decrease of IL-10 were significant in Zishenwan group (Figure 1(d), comparing with either model group or Levofloxacin group). It implicated that Zishenwan enhanced protective immune responses which contributed to the clearance and inhibition of bacteria.

### 3.2. Zishenwan Decreased TLR4 and NF*κ*B Signal in the Mucus

**Figure 2:**
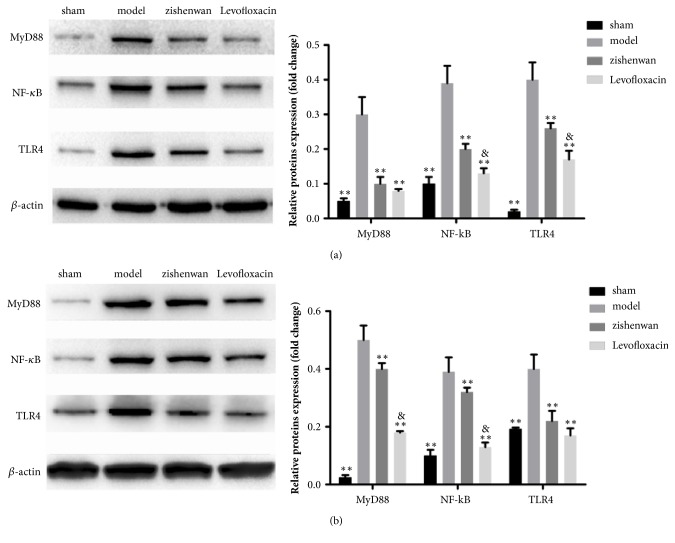
Administration of Zishenwan rescued TLR4-MyD88-NF*κ*B change in rats of chronic pyelonephritis model. (a) In renal pelvis mucosa of model rats, the expressions of TLR4, MyD88, and NF*κ*B/p65 were increased and the administration of Zishenwan significantly decreased the expressions of TLR4, MyD88, and NF*κ*B/p65. (b) In urinary tract mucosa of model rats, the expressions of TLR4, MyD88, and NF*κ*B/p65 were increased and the administration of Zishenwan significantly decreased the expressions of TLR4, MyD88, and NF*κ*B/p65. *∗∗*, p<0.01 compared with model group; &, p<0.05 compared with Levofloxacin group.

Toll-like receptors are important immune receptors which recognize microbial motifs and TLR4 which recognizes LPS is important in chronic pyelonephritis induced tissue damage.* E. coli*, one of gram-negative bacteria family, is rich in LPS, so TLR4 might be activated and participated in the progress of pyelonephritis. We detected the level of TLR4 and its downstream molecular MyD88, and we found the expression of TLR4 and MyD88 significantly increased in the renal pelvis mucosa of model group compared with the sham group (Figure 2(a), p<0.01). Administration of Levofloxacin or Zishenwan significantly decreased the expression of TLR4 and MyD88 (Figure 2(a), p<0.01, comparing with model group). And the expression of TLR4 in the Zishenwan group was much lower comparing with the Levofloxacin group (Figure 2(a), p<0.05) which might relate to the improvement of pathology in the Zishenwan group (Figure 1(b)). Similar results were observed in the bladder mucosa which was implicated by* E. coli* firstly in rat model of chronic pyelonephritis (Figure 2(b)). NF*κ*B, a key factor in cellular responses to stimuli, links to immune response to infection and the secretion of interleukins, but incorrect regulation of NF*κ*B can induce damage of normal cells. In both of renal pelvis mucosa and bladder mucosa, NF*κ*B was kept in a higher level in model group comparing with the sham group (Figures 2(a) and 2(b), p<0.05) which might relate to kidney damage. After treatment, the level of NF*κ*B was decreased in either Levofloxacin group or Zishenwan group (Figures 2(a) and 2(b), p<0.05). According to the above results, treatment of antibiotic or Zishenwan can decrease the activation of TLR4 pathway and NF*κ*B pathway* in vivo*.

### 3.3. Zishenwan Serum Protected Rat Bladder Smooth Muscle Cell from LPS through the Inhibition of TLR4-NF*κ*B Pathways

**Figure 3:**
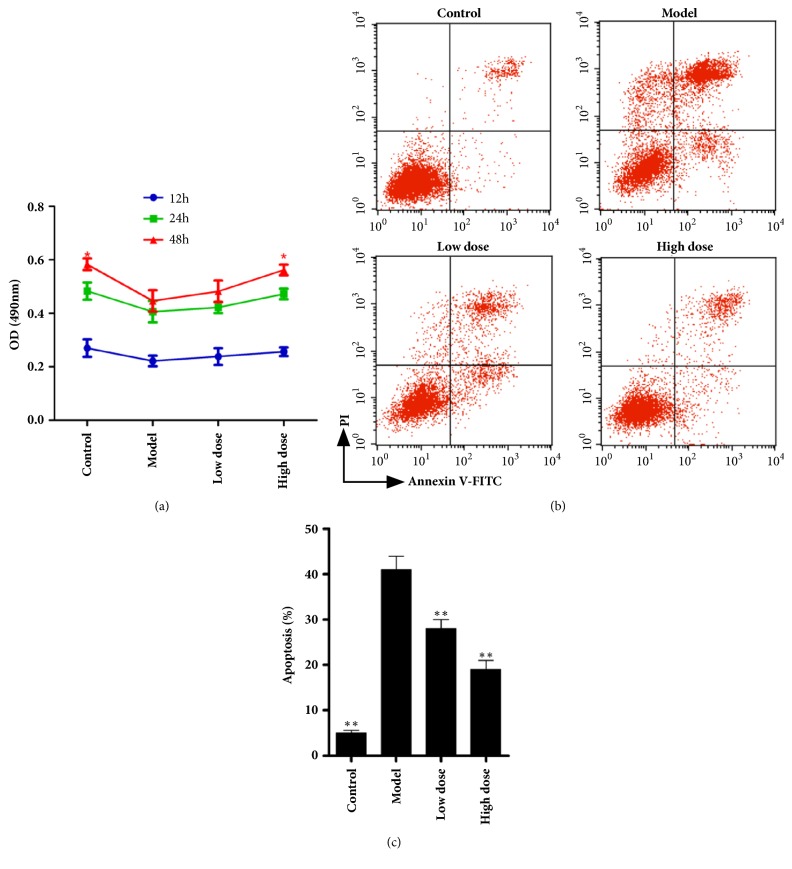
Serum containing Zishenwan protected rat bladder smooth muscle cells from LPS* in vitro*. (a) LPS (10*μ*g/ml) decreased cell viability with time and serum containing Zishenwan rescued rat bladder smooth muscle cells in dose. (b, c) After treated with LPS (10*μ*g/ml) 24 hours, significant apoptosis was induced in rat bladder smooth muscle cells, and the serum containing Zishenwan decreased apoptosis in a dose dependent manner. *∗*, p<0.05 compared with model group; *∗∗*, p<0.01 compared with model group.

From previous results, Zishenwan decreased the expression of TLR4 more effectively than Levofloxacin, and TLR4 expressing in normal cells can induce damage directly. So we speculated that Zishenwan inhibited TLR4 pathway to protect kidney beside the inhibition of infection.* In vitro*, we used LPS (10*μ*g/ml) to induce apoptosis of rat bladder smooth muscle cell and observed protective effects from Zishenwan serum (Figure 3). In the experiment, LPS significantly inhibited growth of rat bladder smooth muscle cell (Figure 3(a), p<0.05, comparing with control group) and induced apoptosis of rat bladder smooth muscle cell* in vitro* (Figures 3(b) and 3(c), p<0.05, comparing with model group). Administration of Zishenwan serum significantly increased the viability of rat bladder smooth muscle cell exposed to LPS in MTT assay and in apoptosis assay in a concentration-dependent manner (Figure 3, p<0.05, comparing with model group).

**Figure 4:**
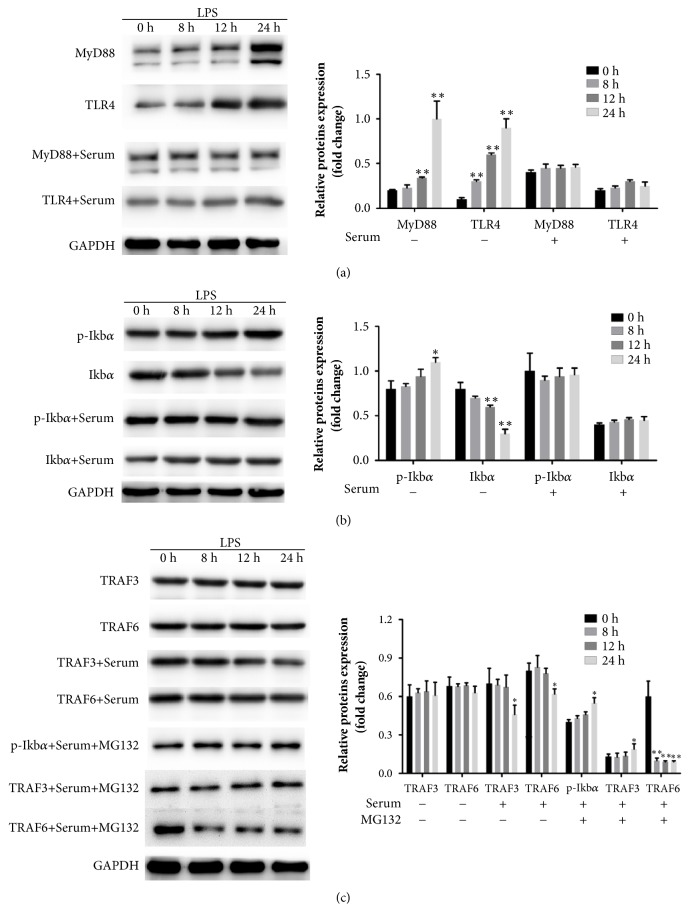
Serum containing Zishenwan stabilized I*κ*B*α* and decreased TRAF3/6, which contributed to the beneficial effect of Zishenwan. (a) LPS induced expression of MyD88 and TLR4 in rat bladder smooth muscle cells and serum containing Zishenwan abolished the increase of both MyD88 and TLR4 (*∗*, p<0.05; *∗∗*, p<0.01 compared with 0 hours). (b) Increased p-I*κ*B*α* level was associated with the decrease of I*κ*B*α* after the incubation of LPS. I*κ*B*α* was stabilized by serum containing Zishenwan and it might accord to p-I*κ*B*α* levels (*∗*, p<0.05; *∗∗*, p<0.01 compared with 0 hours). (c) LPS did not change the expression of TRAF3 and TRAF6, but both of TRAF3 and TRAF6 were decreased after the administration of serum containing Zishenwan. Inhibition of the activation of 20S proteasomes abolished the beneficial changes of p-I*κ*B*α* and TRAF3 mediated by serum containing Zishenwan. (*∗*, p<0.05; *∗∗*, p<0.01 compared with 0 hours).

In western-blot, TLR4 and its downstream molecular MyD88 were significantly increased after the administration of LPS and decreased by Zishenwan serum (Figure 4(a), p<0.05, comparing with LPS group). NF*κ*B was not only the downstream factors of TLR4 but also the activators of transcription. The activation of NF*κ*B relates to the concentration I*κ*B*α*. LPS significantly increased phosphorylation of I*κ*B*α* and decreased I*κ*B*α* (Figure 4(b), p<0.05, comparing with 0 hours). But the administration of Zishenwan serum induced the decrease of p-I*κ*B*α* and induced the increase of I*κ*B*α* (Figure 4(b), p<0.05). Although LPS did not influence the expression of TRAF3 or TRAF6, administration of Zishenwan serum decreased the expression of TRAF3 and TRAF6, important factors activating IKKs (Figure 4(c), p<0.05). The decrease of p-I*κ*B*α* and TRAF3 was abolished by the administration of MG132, a proteasome inhibitor (Figure 4(c), p<0.05). But the treatment of MG132 intensifies the decrease of TRAF6 (Figure 4(c), p<0.01), which might be the result associating with other signals. Zishenwan should inhibit the TLR4 mediated pathway and protected rat bladder smooth muscle cell from LPS induced death. The protective effect comes from the degradation of TRAF3 and TRAF6 which inhibit I*κ*B*α*-mediated TLR4-NF*κ*B signal.

## 4. Discussion

Pyelonephritis is a kidney disease caused by urinary system infection causing serious complications and a few recurrent or delayed renal failures [3, 19]. At present, pyelonephritis is mainly caused by upward infection of a variety of microorganisms, among which the main pathogenic bacterium is* E. coli* [3, 19]. Anti-infective therapies, especially the antibiotics, are major treatment in the clinic, but a variety of adverse reactions and drug resistance also appeared [20]. Another major problem is that immunocompromised patients, such as aged person and child, might be infected repeatedly, and it might induce kidney damage [21, 22]. Traditional Chinese medicines are widely used in clinic treatment of pyelonephritis, especially in China and other Asian countries [4, 5]. Zishenwan is a useful compound Chinese medicinal preparation in clinic, and it is made up of golden cypress*, Anemarrhena asphodeloides *and* Cinnamomum cassia*.* Berberine* in* golden cypress* has been reported to have inhibitory effects on dysentery bacillus,* Staphylococcus aureus*, and other pathogenic bacteria [6].* Anemarrhena asphodeloides* has been reported to have inhibitory effects on* E. coli*, Proteus, and other bacteria [7].* Cinnamon oil* and its main component* cinnamaldehyde* are reported to inhibit growth of Gram negative bacteria and Gram positive bacteria [6, 23]. So, the beneficial effect of Zishenwan was thought to be the antibacterial effect from its components.

But Zishenwan should nourish kidney, clear heat, and clear Qi according to the theory of traditional Chinese medical science which seems different to the antibacterial effect. So we hypothesized that Zishenwan might increase protective effects of immune system and inhibit the tissue damage. Increased secretion of IgA which plays great important role on the resistance to adhering of pathogens to the urinary tract was proved after the administration of Zishenwan. The result boosted our confidence that Zishenwan has other mechanisms benefiting the pyelonephritis patients. From the results, tissue damage was significantly decreased and the effect was much better than the treatment of Levofloxacin. So the direct protection of tissue other than antimicrobials effect should exist in the treatment of Zishenwan.

Gram negative bacteria are major pathogenic microorganisms and they are rich in LPS, a key ingredient inducing tissue damage. TLR4, an important receptor of LPS, was increased in urethral mucus of model rats. And the activation of TLR4 increases immune injury and induces direct tissue damage [16, 17]. Meanwhile, secretions of interleukins and NF*κ*B which controls transcription of interleukins were activated in urethral mucus of model rats. After the treatment of Zishenwan inhibited the activations of TLR4 pathway and NF*κ*B were inhibited, and interleukins related to tissue damage were also decreased. This result was also proved* in vitro*. So Zishenwan can decrease Gram negative bacteria induced tissue damage by immune regulation.

From our results, Zishenwan can protect kidney from damage induced by LPS by the inhibition of TLR4 related pathway and it is other than the direct antibacterial activity. This effect is more consistent with theory of traditional Chinese medical science and it might connect the traditional Chinese medical science with modern biological theory.

## Data Availability

The data used to support the findings of this study are available from the corresponding author upon request.
